# Disease and Drug Action

**Published:** 1897-11

**Authors:** W. S. Talcott


### DISEASE AND DRUG ACTION.

W. S. Talcott, M. D.

While much has been written advancing various theories explanatory of medicinal drug action, more especially in the dilute preparations, it now appears that the only tenable theory is the energistic—this accords with present scientific theory as to the persistence of things—that is, the universe—and surely mankind is part.

Every medicinal drug embodies a distinctive force—energy—which can be transferred to other substances, and by the law of semiology applied to the healing of the sick—that is, to restore the correlation of forces—energies—lost in the sick.

In functional cases correlation established, health is restored, where pathological lesions exist consequent physiological action heals the lesion.

The impression of the exact homœopathic prescription is psychological, resulting in physiological change.

This indicates that the only medicinal action of a drug is its direct or primary action, and that the more dilute the dose that cures the more stable the cure.

The purpose is to disabuse the mind of the mechanical theory of things, enlarge the scope of expectation in regard to drug action and lead to purer practice.

Dr. Baylies—The first proposition in the theory of "disease and drug action," formulated by Dr. W. S. Talcott, that "medicinal drug action is energistic," is demonstrated by all experience with the higher potencies, and consistent with Hahnemann's ascription to every medicine of a peculiar spirit-like force; and by the fact that such forces are susceptible of potential development in degrees thus far unlimited; that they are transferable to other substances, and even by contact diffusible, throughout a neutral vehicle, as from medicated through unmedicated pellets, by Korsakoff's method; and their healing powers are proved when applied through such media upon the sick.

In the second proposition Dr. Talcott refers to the homœopathic law for their application: It is the all-embracing life-force which maintains the correlation of the elementary forces of the body, and its disturbance or disorder which destroys their normal correlation and constitutes sickness. The explanation of medicinal action given by our learned friend, Dr. Fincke, according to the Newtonian law of motion, may be correct, and that the inter-action of the suitable medicine and the disturbed life-force is that of two forces of which the action and reaction are equal and contrary; the contrary is the resultant remedial action.

Regarding the fourth proposition in Dr. Talcott's formula it seems that the "law of the similars" would necessitate rather a primary action of the remedy upon the life-force, or its "*components,*" the elementary forces of the body, than directly upon the psychic principle; and that the psychical phenomena produced by every medicine are secondary.

Dr. Baylies also presented Dr. Fincke's commentary, which follows:

BROOKLYN, October 22d, 1897.

B. L. B. BAYLIES, M. D.:

DEAR DOCTOR:—I return enclosed Dr. Talcott's paper with thanks, and add a few remarks which may clear up some obscure points on it and bring down the matter to a simple chapter in natural science. I at least understand Hahnemann in this way.

See also my article "Dynamics of Homœopathy and Natural Science;" the article of J. H. Allen in the last meeting, my commentary on antipathy, direct and inverse action, etc.

Yours fraternally, B. FINCKE.

